# Operational challenges and adaptive leadership in emergency departments in the United States of America: a mixed-methods analysis

**DOI:** 10.1186/s12873-025-01400-y

**Published:** 2025-11-19

**Authors:** Emmanuel Animashaun, Ellen Barnie Peprah, Olaoluwa Olorunfemi, Olufunmike Oyekunle, Emmanuel Nortey-Adom, Sharon Karbo

**Affiliations:** 1 Department of Health Policy and Management, Johns Hopkins Bloomberg School of Public Health, Baltimore, MD USA; 2 Johns Hopkins Bloomberg School of Public Health, Baltimore, MD USA; 3 Bowen University Teaching Hospital, Ogbomoso, Nigeria; 4 University of Ilorin Teaching Hospital, Ilorin, Nigeria

**Keywords:** Emergency department, Operational challenges, Healthcare leadership, Mixed-methods, Rural health, Staffing models, Triage, Adaptive leadership, Interprofessional collaboration, Quality improvement

## Abstract

**Background:**

Emergency departments (EDs) are important access points for acute care in the U.S. healthcare system. However, persistent operational challenges, ranging from overcrowding to staffing shortages continue to threaten care quality and provider well-being. While existing literature has explored patient-level outcomes and system bottlenecks, the perspectives of ED leadership remain underexamined.

**Objective:**

To explore how ED leaders across diverse facility types perceive, prioritize, and respond to operational challenges, and to identify context-sensitive strategies for improvement.

**Methods:**

We employed a sequential explanatory mixed-methods design, combining survey data (*n* = 40) with semi-structured interviews (*n* = 8) of ED leaders representing rural, urban, and academic settings. Quantitative data were analyzed descriptively, while qualitative data underwent thematic analysis. Findings were triangulated to identify patterns across five operational domains: capacity management, staffing models, care coordination, digital integration, and patient experience.

**Results:**

Leaders expressed near-universal satisfaction with triage protocols (94%) and onboarding practices (76%), signaling possible successful standardization. However, significant role-based and contextual divergences emerged, particularly around staffing adequacy, handoff quality, and revenue cycle awareness. Rural leaders reported greater innovation despite resource constraints, leveraging simplified protocols and creative staffing models. Strategic priorities such as space optimization and technology integration were often misaligned with operational realities, highlighting systemic implementation barriers.

**Conclusion:**

ED leadership effectiveness hinges on adaptive strategies tailored to local contexts. While certain practices can be standardized, many require customization based on facility type, leadership role, and resource availability. These findings support a differentiated approach to ED improvement, one that balances evidence-based protocols with entrepreneurial adaptability and cross-professional collaboration.

**Clinical trial number:**

Not applicable.

**Supplementary Information:**

The online version contains supplementary material available at 10.1186/s12873-025-01400-y.

## Introduction

Emergency departments (EDs) serve as vital nodes in health systems, acting as round-the-clock entry points for individuals requiring unscheduled, urgent, or emergent care. In the United States, EDs handle over 150 million visits annually, representing a key component of care access for medically complex, socially vulnerable, and underinsured populations [1–3]. In recent times, the roles of EDs have expanded beyond traditional emergency care, with emerging areas like behavioral health triaging [4, 5], syndromic surveillance [6, 7], and disaster response [3, 8], underscoring their evolving importance in modern healthcare delivery.

Despite their critical role, emergency departments face mounting operational pressures that threaten patient safety, extend wait times, and contribute to provider burnout [9–11]. Over the past two decades, extensive research has documented these challenges, with studies predominantly examining patient-level outcomes such as length of stay, readmission rates, and satisfaction scores, or analyzing specific operational bottlenecks like triage delays and bed turnover inefficiencies [10, 12]. While this literature has generated valuable insights into the scope and consequences of ED operational dysfunction, it has largely overlooked the perspectives of those responsible for implementing solutions: ED leadership teams.

This gap is particularly significant given that ED leaders, including physician leaders, nurse leaders, and administrative supervisors serve as the critical bridge between organizational policy and frontline practice, and must translate institutional directives into actionable workflows, adapt to evolving regulatory requirements, and sustain performance improvements amid resource constraints and staff turnover [13, 14]. Yet empirical research examining how ED leaders navigate these responsibilities, prioritize competing demands, and perceive barriers to operational improvement remains limited.

Moreover, the existing literature has inadequately addressed how organizational context shapes leadership experiences and decision-making processes. Emergency departments operate within vastly different settings, from resource-constrained rural facilities serving broad geographic areas to high-volume urban safety-net hospitals and well-resourced academic medical centers with resident coverage and subspecialty support [15–17]. These contextual differences likely influence how leaders experience operational challenges, adopt workflow innovations, and allocate limited improvement resources, yet few studies have systematically examined these variations. Understanding these leadership perspectives and contextual differences is essential for developing more effective, tailored approaches to ED operational improvement that account for the realities of implementation rather than focusing solely on outcomes measurement.

While this study focused on U.S. emergency departments, the operational challenges examined represent fundamental healthcare delivery issues that transcend national boundaries [18]. Health systems globally, particularly in low- and middle-income countries, often face similar pressures of overcrowding, staff burnout, and resource limitations, though typically with fewer technological and financial resources to address them [19]. Leadership strategies for workflow optimization, team communication protocols, and adaptive resource allocation developed in well-resourced U.S. settings may offer scalable insights for healthcare administrators worldwide.

This study addressed these research gaps by examining how U.S. ED leaders perceived, prioritized, and responded to operational challenges across five key domains: capacity management and patient boarding; staffing models and workforce retention; shift handoffs and care team communication; documentation systems and digital integration burden; and patient satisfaction and safety. The five domains were developed by the research team based on extensive quality improvement consulting experience across diverse emergency departments in the United States and analysis of recurring themes from multiple ED performance consulting reports. These domains align with established performance priorities in emergency care literature and were refined through iterative feedback from ED leaders and stakeholders during survey development and pilot testing. While the domains are presented as discrete categories, they reflect overlapping and interdependent aspects of emergency department operations. For instance, patient satisfaction and safety encapsulates frontline indicators of care quality and includes both patient experience metrics and safety-related concerns such as care delays, communication breakdowns, and adverse events. Rather than treating quality of care as a standalone category, we operationalized it through the lens of safety and satisfaction, which ED leaders consistently identified as tangible and actionable proxies for broader quality performance. This integrative approach reflects the way frontline leaders typically monitor and intervene in quality issues, balancing measurable patient outcomes with operational levers under their direct influence.

Our findings contribute to the growing body of literature on healthcare leadership and operational resilience while providing actionable insights for ED administrators and health system leaders.

## Methods

### Study design

We employed a sequential explanatory mixed-methods design to systematically examine emergency department leadership perspectives on predefined operational challenges while capturing contextual nuances through qualitative exploration. This approach enabled quantification of priority challenges and satisfaction levels through survey data while capturing the contextual nuances, implementation experiences, and underlying reasoning through in-depth interviews [20].

We acknowledge that a sequential exploratory design might have allowed operational challenge domains to emerge naturally from qualitative data first. However, our explanatory approach was justified by several factors: (1) the extensive consulting experience base documenting consistent ED operational challenges across diverse facilities; (2) the need for systematic comparison of leadership perspectives across different facility types and roles; and (3) the goal of quantifying satisfaction and priority levels within established operational domains before exploring implementation experiences and contextual factors.

While this design means we provided rather than discovered key challenge domains, our priority ranking exercises and open-ended survey responses demonstrate that leader priorities aligned closely with our consulting-derived framework, suggesting the predefined domains captured authentic leadership concerns while enabling systematic cross-contextual analysis.

### Participants and recruitment

Emergency department (ED) leaders were recruited through direct outreach to a non-random, convenience sample of professional network spanning diverse U.S. healthcare settings. Email invitations were sent to 500 individuals holding formal leadership roles such as medical directors, Chief Nursing Officers (CNOs), and ED directors across varied facility types and geographic regions. To ensure relevance to operational complexity, only leaders from EDs with annual patient volumes exceeding 10,000 visits were eligible to participate. Three reminder emails were sent at two-week intervals following the initial invitation. A total of 40 leaders completed the survey, yielding a response rate of 8%. While modest, this response rate aligns with expected trends for executive-level healthcare professionals, who often face significant time constraints and frequent research requests.

Given the exploratory and descriptive nature of this mixed-methods study, sample size was not formally calculated and was determined based on feasibility, diversity of perspectives, and the need to enable qualitative follow-up. This pragmatic approach is consistent with standards in health services and implementation research where statistical generalizability is not the primary aim.

All 40 survey respondents were invited to indicate interest in participating in follow-up interviews. Of the leaders who indicated interest, eight were invited to follow-up interviews to represent diverse geographic regions, facility types (academic, rural, and community), and leadership roles. While this represents a convenience sample rather than purposive selection, the resulting participant diversity aligned well with maximum variation sampling principles commonly used in qualitative inquiry.

### Survey and interview instrument development and validation

The 19-item survey instrument was systematically developed through a multi-stage process. First, we conducted a comprehensive literature scan to identify operational challenge domains prevalent in emergency medicine research. Second, the instrument was validated through expert consultation with two senior emergency department consultants, one physician and one nurse, each with extensive operational leadership experience. The final instrument comprised Likert-scale, ranking, and multiple-choice questions across five domains: (1) capacity management and patient boarding, (2) shift handoffs and care coordination, (3) staffing models and workforce retention, (4) patient safety and satisfaction outcomes, and (5) documentation systems and digital integration burden. Demographic variables captured respondent role, facility characteristics, geographic region, and trauma designation.

Semi-structured interview questions were developed through a systematic process directly connected to survey findings. Interview questions were designed to: (1) explore the “why” behind survey response patterns, (2) understand implementation experiences related to operational challenges, and (3) capture contextual factors influencing leadership decisions. Question development followed three stages: First, we analyzed preliminary survey data to identify areas requiring deeper exploration (e.g., role-based differences in satisfaction ratings, strategic-operational disconnects). Second, we developed open-ended questions targeting these areas, focusing on workflow optimization strategies, innovation implementations, and barrier identification. Third, we pilot-tested questions with 2 ED leaders not included in the main study to ensure clarity and relevance. The interview guide included core questions about: operational improvement strategies, successful innovation examples, interprofessional collaboration experiences, and systemic barriers to change. Probing questions were tailored based on participants’ survey responses, creating direct survey-interview integration while maintaining consistency across interviews.

### Data collection

Survey data were collected online via Qualtrics between January and February 2024, yielding forty completed responses. The survey instrument is available as Supplementary File 1.

Semi-structured interviews were conducted via secure videoconferencing between March and April 2024, lasting 30–45 min each (see supplementary file 2). Eight interview participants were selected to ensure geographic diversity and representation across facility types. Interview topics explored workflow optimization strategies, successful innovation implementations, community partnership development, and systemic barriers to operational improvement. All interviews were digitally recorded with participant consent and transcribed verbatim.

### Data analysis

Quantitative survey data were analyzed using descriptive statistics, including frequencies, proportions, and cross-tabulations by respondent characteristics and facility types. Given the exploratory nature of this study and sample size considerations, we focused on identifying meaningful patterns and trends rather than formal hypothesis testing. Open-ended survey responses underwent directed content analysis guided by our five operational domains framework. This approach allowed systematic categorization of responses using our pre-defined domains as initial coding categories while remaining open to emergent themes and novel concepts not captured by the predefined framework.

Qualitative interview data were analyzed using inductive thematic analysis. Two researchers independently coded all transcripts manually, with initial codes developed through line-by-line analysis. Codes were systematically organized into potential themes, which were reviewed and refined through iterative discussion. Final themes were established through researcher consensus and member checking with selected participants.

Finally, data triangulation was employed to identify convergence, divergence, and elaboration between survey and interview findings, enhancing the overall validity of our results.

### Ethical considerations

This study was determined by the Institutional Review Board at the Johns Hopkins Bloomberg School of Public Health not to qualify as human subjects research under the U.S. Department of Health and Human Services regulations (45 CFR 46.102). As such, the study did not require IRB oversight. Ethical practices were followed in all interactions, and data were de-identified to ensure confidentiality. No identifiable private information was collected or analyzed.

## Results

Of the 500 emergency department leaders invited to participate, 40 completed the online survey, yielding an 8% response rate. Among these, 35 respondents provided at least one free-text comment in open-ended questions, generating 124 unique meaning units. These were grouped into 17 sub-categories and further clustered into five overarching thematic categories that aligned closely with our predefined operational domains.

In the qualitative phase, eight leaders participated in semi-structured interviews. Thematic analysis of interview transcripts yielded 312 meaning units, which were coded into 27 sub-categories and synthesized into six major themes that provided deeper contextual understanding of leadership experiences and implementation strategies.

The following results integrate both survey and interview data to examine patterns of consensus, divergence, and adaptation in emergency department leadership perspectives across diverse healthcare settings.

### Sample characteristics

Table 1 presents the demographic and facility characteristics of the survey respondents, highlighting diversity across geographic regions and leadership roles. Forty emergency department leaders completed the survey, representing diverse U.S. healthcare settings. The majority (69%) possessed over 10 years of leadership experience, with nursing leaders comprising 68% of respondents and physician leaders 32%. Community hospitals dominated the sample (53%), followed by academic medical centers (21%) and rural emergency hospitals (13%). Facilities ranged from small rural hospitals (< 100 beds, 32%) to large academic centers (500 + beds, 21%), with 65% designated as trauma centers and patient volumes spanning 10,000 to over 50,000 annual visits.

**Table 1:** Sample demographics and facility characteristics (*N* = 40)

Characteristics	%
**Years of leadership experience**	
1–5 years	20
6–10 years	10
11–15 years	30
16–20 years	25
> 20 years	15
**Leadership Role**	
ED Director (Nurse)	50
Chief Nursing Officer (Nurse)	18
ED Medical Director (Physician)	15
ED Manager (Physician)	17
**Facility Type**	
Community Hospital	53
Academic Hospital	21
Rural Emergency Center	11
Integrated Delivery Network	11
Critical Care	6
**Trauma Center Designation?**	
Yes	65
No	35
**Annual ED Visit Volume**	
10,000–50,000	68
50,000+	32
**Bed Size**	
Under 100	32
100–500	47
500+	21

### Leadership consensus: operational domains with universal success

Despite diverse settings and roles, two operational areas achieved remarkable leadership alignment, suggesting mature practices where standardized approaches have succeeded.

#### Triage operations as foundation

Survey data revealed 94% of ED leaders rated their triage protocols as effective – the highest satisfaction score across all operational domains. This consensus transcended facility types, leadership roles, and geographic regions. While we did not directly assess the content or uniformity of triage protocols across sites, this consistently high satisfaction may indicate the presence of established practices perceived to be working well. Several interviewees also described triage as a well-oiled process requiring minimal troubleshooting. As one respondent noted, *“That’s the part of the ED we rarely have to fix*,* our triage nurses know exactly what to do.”* Another rural leader summarized the widespread experience: *“Our triage system just works*,* it’s the one thing we never worry about.”*

#### Commitment to comprehensive onboarding

Three-quarters (76%) expressed satisfaction with onboarding processes, reflecting philosophical commitment despite operational challenges. Rural leaders particularly emphasized dedication despite constraints: *“We don’t short our staff on orientation… anybody that comes into the ER gets at least a month.”* However, the 24% reporting dissatisfaction concentrated among facilities facing systemic barriers, where one nursing leader noted improvement from *“six months to get a person onboarded… down to about two and a half [months]… but two and a half when you’re desperate for staff seems a long*,* long time.”*

### Role and context-driven divergences

While consensus emerged in standardized domains, significant differences appeared based on leadership roles and organizational contexts, revealing how professional backgrounds shape operational perspectives.

#### Shift handoffs: professional role divide

Table 2 summarizes satisfaction ratings across the five operational domains, identifying areas of alignment and divergence across leadership roles and facility types. One-third (33%) rated handoff procedures as neutral or ineffective, and nursing leaders consistently indicated greater dissatisfaction than physicians. This may reflect nursing leaders’ direct responsibility for workflow coordination. Successful programs transcended role differences through systematic implementation, with rural facilities often achieving superior results through simplified approaches: *“Every shift change… they give handoff right at the desk… because we’re such a small unit that information is readily passed on.”*

**Table 2:** Effectiveness rating of shift handover procedures

Rating	Frequency
Very Ineffective	1 (3%)
Ineffective	1 (3%)
Neutral	9 (27%)
Effective	17 (52%)
Very Effective	5 (15%)

#### Staffing adequacy through different lenses

While 74% rated staffing alignment as satisfied, physician leaders showed markedly higher satisfaction (85%) compared to nursing leaders (63%), a 22-percentage-point gap suggesting fundamentally different workforce perspectives. The distribution of concerns reinforced this divide: 67% of staffing dissatisfaction came from ED Directors versus 33% from Medical Directors, with 56% concentrated among smaller facilities serving fewer than 50,000 annual patients.

#### Revenue cycle awareness gaps

The starkest role-based divergence emerged in revenue cycle awareness. All physician leaders (100%) acknowledged experiencing denied claims and coding issues, compared to nursing leaders where 45% reported frequency as “unknown.” This pattern reflects different operational exposure, with physicians more directly involved in documentation decisions affecting billing outcomes.

#### Rural-urban operational divides

Rural leaders operated within fundamentally different constraints that shaped entire approaches to care delivery. Transport logistics dominated decision-making: *“Most of our super critical patients… will fly out… it’s about a three-hour drive… but it’s probably safer and more efficient to fly.”* These dependencies created different success metrics, with rural leaders prioritizing specialty access over throughput optimization, while urban leaders focused on volume management and technology integration.

### Strategic-operational disconnects

Analysis revealed significant gaps between leaders’ strategic priorities and current operational satisfaction, indicating areas where resource allocation may need realignment.

#### Physical space: the universal challenge

Leaders overwhelmingly ranked optimized physical space as their highest improvement priority, yet this urgency wasn’t reflected in satisfaction ratings. This disconnect suggests space constraints represent a known but unaddressed systemic issue. One nursing leader described extreme conditions: *“I have 66 total beds in my ED… 90% filled with boarders… It leaves me [with] 11 beds… to see emergency department patients.”*

#### Technology integration: aspiration meets reality

Despite ranking AI and virtual technology as high priorities, most facilities lacked implementation capabilities. This gap was particularly pronounced in rural settings, though leaders expressed clear vision: *“I think AI and machine learning will be huge… not to do our work for us*,* but to really act as a quality checker.”* Progressive facilities demonstrated innovation potential: *“It’s updating you on your care… it gives power back to the patient where they feel like people haven’t forgotten about them.”*

#### Revenue cycle: the administrative disconnect

While 67% reported occasional or frequent revenue cycle issues, this operational challenge ranked lowest among improvement priorities. This suggests revenue cycle management may be perceived as administrative rather than clinical leadership responsibility, despite direct operational impact.

### Adaptive leadership strategies

Leaders who reported implementing effective operational improvements demonstrated remarkable adaptability, developing innovative solutions within resource constraints through necessity-driven innovation.

#### Operational innovation under pressure

Resource constraints drove creative solutions. One nursing leader transformed their care model: *“We changed our staffing model… we have a provider on triage*,* and we built up our nursing team… a first look nurse*,* a treatment nurse*,* and then another nurse that rounds through the department.”* This provider-in-triage model represents fundamental workflow reimagining.

#### Educational leadership philosophy

Successful leaders emphasized educational over punitive approaches, transcending facility types. One experienced leader explained: *“I always start with educational opportunity… I’m going to assume you don’t know. And so*,* therefore*,* I will teach you… And then if you choose not to use the education*,* then it becomes disciplinary.”*

Together, these findings demonstrate that while ED leaders face common challenges, their responses vary significantly based on professional role, facility characteristics, and geographic context. Successful strategies consistently emerged from local adaptation rather than standardized approaches, suggesting effective emergency care leadership requires both operational expertise and entrepreneurial adaptability.

## Discussion

Our findings reveal that effective emergency department leadership requires context-specific adaptation rather than standardized approaches, challenging prevailing assumptions about uniform best practices in ED operations.

### Navigating standardization and adaptability: when universal solutions work and when they don’t

The near-universal satisfaction with triage protocols (94% satisfaction) contrasts sharply with the significant divergences observed in other operational domains, offering important insights into the dynamics of healthcare operations. Triage protocols appear to represent a widely adopted practice where established algorithms have enabled consistent implementation across diverse settings with limited need for local modification.

By contrast, domains like shift handoffs and staffing models demonstrated substantial variation across contexts, reflecting how local operational, cultural, and capacity-related factors shape how practices are deployed. This finding emphasizes the need for contextual adaptability in these domains, in line with research demonstrating that failure to consider local contextual factors can lead to failure of transfer of healthcare quality improvement programs to new settings [16, 20, 21].

The success of simplified handoff approaches in rural settings compared to more complex systems in larger facilities further supports this distinction. Rural leaders achieved superior outcomes through basic communication protocols, while larger facilities with sophisticated tools sometimes struggled with implementation consistency. This paradox reflects broader organizational literature on the trade-offs between procedural complexity and execution reliability [22].

Our findings suggest that some operational strategies such as triage protocols may benefit from widespread standardization, while others, particularly those involving staffing logistics or interdisciplinary communication, require greater flexibility to account for local realities. Improvement strategies should therefore distinguish between practices that are inherently portable and those that require deliberate contextual adaptation to succeed [23, 24].

### Professional role as operational lens: the physician-nursing leadership divide

The systematic differences between physician and nursing leaders’ perspectives on staffing adequacy and revenue cycle awareness represent more than simple disagreement; they reflect fundamentally different operational vantage points that shape problem identification and solution prioritization. This finding extends interprofessional collaboration literature by demonstrating how professional roles create distinct leadership perspectives even when managing the same operational challenges [25–27]. Healthcare professionals view problems differently and from different perspectives because their professions are based on different knowledge traditions, and this creates complexity in interprofessional collaboration processes [28].

Nursing leaders’ consistently lower satisfaction with staffing and handoff procedures likely reflects their direct responsibility for workflow coordination and shift coverage decisions. Physician leaders’ universal awareness of revenue cycle issues compared to nursing leaders’ frequent “unknown” responses (45%) suggests clear differences in operational exposure rather than knowledge gaps. These role-based perspectives have important implications for improvement team composition and intervention design. The divergence highlights potential blind spots in traditional ED leadership structures. If physician leaders consistently perceive adequate staffing while nursing leaders experience shortages, improvement initiatives led primarily by physicians may inadequately address frontline workforce challenges. Similarly, revenue cycle improvements may lack nursing leader buy-in if these leaders feel disconnected from billing-related decisions that affect their operational responsibilities.

This professional divide parallels findings in other healthcare settings where physicians and nurses demonstrate different priorities and problem perceptions [26, 29, 30]. These differences may however, be particularly pronounced in emergency departments where rapid decision-making may amplify the consequences of misaligned leadership perspectives.

### Rural constraints as catalysts for creativity

Rural ED leaders’ fundamentally different operational realities – transport logistics, specialty access limitations, and resource constraints – drove innovative solutions that challenge assumptions about optimal emergency care delivery. The success of simplified communication protocols and creative staffing models in rural settings demonstrates how resource constraints can catalyze rather than impede operational excellence, consistent with “frugal innovation” literature from resource-constrained healthcare environments [31, 32]. Frugal innovation encompasses heterogeneous activities providing effective functional solutions to common problems with minimal use of resources, and innovations frequently arise in low-resource settings when usual solutions are too expensive or not available [31].

The rural leaders’ focus on specialty access and transfer capabilities over throughput optimization represents a context-appropriate redefinition of success metrics. This finding has important implications for quality measurement and improvement initiatives, which typically emphasize standardized metrics like length of stay and patient satisfaction that may not capture the most critical performance indicators for rural emergency departments [16, 33].

Urban leaders’ emphasis on volume management and technology integration reflects different operational pressures but may also suggest missed opportunities for adaptation strategies that rural leaders have developed out of necessity. The innovative provider-in-triage model described by one nursing leader represents the type of fundamental workflow reimagining that could benefit facilities across contexts, yet such innovations may remain isolated without systematic knowledge sharing mechanisms.

Rural healthcare settings face unique constraints including technology adoption delays, with rural leaders consistently reporting implementation lags five to ten years behind urban centers [16, 34]. This technology lag may create a bifurcated landscape where urban facilities implement advanced workflow solutions while rural facilities rely on more fundamental operational improvements, yet rural settings may develop creative alternatives that offer lessons for all healthcare environments.

### Strategic-operational implementation gaps: when priorities don’t drive practice

The disconnects between stated improvement priorities and current operational satisfaction reveal systemic barriers to translating strategic vision into operational reality. Leaders’ overwhelming emphasis on physical space optimization despite this not being reflected in satisfaction ratings suggests that space constraints represent a known but structurally unaddressed challenge requiring hospital-level rather than department-level solutions.

Similarly, the gap between technology aspiration and implementation capability, particularly pronounced in rural settings, reflects broader healthcare technology adoption patterns where resource availability determines implementation timelines regardless of clinical need or leader vision [31]. The five-to-ten-year technology lag reported by rural leaders has implications for health equity, as these delays may compound disparities in care quality and efficiency.

The revenue cycle disconnects where 67% experience problems but rank this lowest for improvement suggests these issues may be perceived as administrative rather than operational leadership responsibilities. This perception gap could impede comprehensive approaches to ED operational improvement, as revenue cycle efficiency directly affects resource availability for other improvement initiatives.

### Limitations

Several limitations constrain the generalizability and interpretation of these findings. The modest sample size (*n* = 40) and recruitment through professional networks may introduce selection bias toward more engaged or experienced leaders. The small interview sample size (*n* = 8) is also a limitation. While the participants represented diverse facility types and roles, the limited number may constrain the generalizability of qualitative insights. The cross-sectional design limits causal inference about the relationship between contextual factors and leadership perspectives. Self-reported data may be subject to social desirability bias, particularly regarding operational satisfaction ratings. Our use of predefined operational domains in the survey, though enabling structured comparison, may have constrained the emergence of novel insights. However, the inductive analysis of interview data helped surface context-specific themes beyond the original framework.

Also, the U.S.-specific context may limit applicability to healthcare systems with different organizational structures, payment models, or regulatory environments. Additionally, the study period (January-April 2024) represents a single time point that may not capture seasonal variations or longer-term trends in operational challenges and leadership responses.

### Future research directions

These findings suggest several productive directions for future research. Longitudinal studies tracking how leadership perspectives and improvement strategies evolve over time could provide insights into successful adaptation processes. Larger-scale validation studies could confirm the role-based and contextual differences identified here while exploring additional factors that influence leadership perspectives.

International comparative studies could examine whether similar patterns emerge in different healthcare systems, potentially identifying universal versus system-specific leadership challenges. Implementation science studies focusing on successful contextual adaptations could provide more detailed guidance for translating effective strategies across different settings.

Finally, intervention studies testing context-specific improvement approaches compared to standardized interventions could provide empirical evidence for the adaptive leadership model suggested by these findings.

## Practical implications for emergency department leadership and policy

Our findings offer actionable insights for ED leaders and health system administrators seeking context-sensitive operational improvements.

### Differentiating between standardized and contextual practices

While triage protocols have achieved near-universal satisfaction, other operational areas like shift handoffs and staffing models exhibit significant variability. This suggests the need to distinguish between operational elements suitable for standardization and other elements requiring local customization. Implementing this differentiation can improve the effectiveness of quality improvement initiatives by ensuring that standardized protocols are applied where appropriate, and flexibility is maintained where necessary.

### Fostering interprofessional collaboration in leadership

The observed disparities between physician and nursing leaders regarding staffing adequacy and revenue cycle awareness highlight the importance of diverse perspectives in leadership. Incorporating both clinical and administrative viewpoints can lead to more comprehensive decision-making processes. Establishing interprofessional leadership teams can bridge gaps in understanding and ensure that operational strategies address the concerns of all stakeholders.

### Leveraging rural innovations across settings

Rural EDs have demonstrated innovative approaches to operational challenges, such as simplified communication protocols and creative staffing models. These “frugal innovations” can offer valuable insights for urban and resource-rich settings. Encouraging knowledge exchange between rural and urban EDs can facilitate the adoption of effective practices across diverse healthcare environments.

### Aligning strategic priorities with operational realities

The disconnect between strategic goals (e.g., technology integration) and operational satisfaction indicates a need for better alignment. Conducting regular assessments to evaluate the feasibility of strategic initiatives can help ensure that resources are allocated effectively and that implementation plans are grounded in the current operational context.

### Enhancing revenue cycle management awareness

The lack of awareness among nursing leaders regarding revenue cycle issues suggests a need for broader education and involvement in financial processes. Providing training and fostering collaboration between clinical and financial departments can improve revenue cycle management and ensure that operational decisions are informed by financial considerations.

### Investing in leadership development programs

Adaptive leadership is crucial for navigating the complexities of ED operations. Investing in leadership development programs that focus on resilience, innovation, and interprofessional collaboration can equip leaders with the skills necessary to drive continuous improvement and respond effectively to emerging challenges.

## Conclusion

Emergency departments occupy a critical space in health systems, but operational challenges continue to strain their capacity to deliver timely, safe, and equitable care. This study underscores that while some solutions, like triage protocols, may benefit from standardization, many operational domains demand context-specific adaptation. Leadership perspectives vary not only by role but also by setting, shaping how problems are understood, and solutions are enacted. Recognizing the value of local innovation, interprofessional collaboration, and strategic-operational alignment is essential for sustainable improvement. By illuminating these dynamics, this study contributes to a more nuanced understanding of emergency department functionality and offers a practical framework for adaptive leadership in complex healthcare environments.

## Supplementary Information

Below is the link to the electronic supplementary material.

Supplementary Material 1

Supplementary Material 2

## Data Availability

The datasets generated and/or analyzed during the current study are not publicly available due to participant confidentiality and institutional restrictions but are available from the corresponding author on reasonable request.

## Appendix A: Glossary of terms

**Boarder**: Patient who has been admitted to the hospital but remains physically in the emergency department due to lack of available inpatient beds.

**CNO**: Chief Nursing Officer - senior executive responsible for nursing practice across the healthcare organization.

**ED Director**: Senior nursing leader responsible for emergency department operations, staffing, and quality improvement.

**Medical Director**: Physician leader responsible for clinical protocols, medical staff oversight, and quality assurance.

**Provider**: Healthcare clinician (physician, nurse practitioner, or physician assistant) authorized to make clinical decisions and prescribe treatments.

**Revenue Cycle**: Financial processes including patient registration, insurance verification, billing, and payment collection.

**Safety-net Hospital**: Healthcare facility providing care regardless of patients’ ability to pay, often serving uninsured and underinsured populations.

**Trauma Designation**: Official recognition (Level I-IV) indicating a hospital’s capability to provide comprehensive trauma care.

**Triage Protocols**: Systems or guidelines used to prioritize patient treatment based on the severity of their condition.
