# Supplementary material for: Operational challenges and adaptive leadership in emergency departments in the United States of America: a mixed-methods analysis
Source: BMC Emerg Med. 2025 Nov 19;25:241. doi: 10.1186/s12873-025-01400-y (PMC12628554; doi:10.1186/s12873-025-01400-y)
Supplement: Supplementary file 1 — Supplementary Material 1 [file 12873_2025_1400_MOESM1_ESM.docx]

POWERING ED PERFORMANCE SURVEY

Thank you for taking the time to participate in our survey. Your insights will be pivotal in our efforts to enhance the performance of emergency departments across the United States. By sharing your experiences, challenges, and suggestions, you play a vital role in shaping solutions that will directly address the needs of emergency departments around the country.

**About us** We are a team of Master of Business Administration (MBA) students from the Johns Hopkins University - Carey Business School, taking a class in Foundations of Business of Health. The class provides an overview of the evolution, structure and current issues in the health care system by examining three broad segments of the health care industry: payors, providers and suppliers.

**Survey Duration:** This survey is expected to take approximately 1-3 minutes to complete.

**Confidentiality and Privacy:** Your responses are completely confidential, and your individual information will not be shared. All data collected will be used solely for research purposes. By checking the circle below, you confirm that your responses be used for the purposes stated above.

If you agree, please sign here………………………….

**Part A.**

1. Your Role

- Chief Nursing Officer
- ED Director
- ED Manager
- ED Medical Director
- Other

2. If you selected other, please enter role here.

________________________________________________________________

3. Years of leadership experience

- 0-3 years
- 3-5 years
- 5-10 years
- 10 years+

4. Type of hospital

- Integrated Delivery Network (IDN)
- Academic
- Community
- Critical care
- Rural emergency hospital
- Other

5. If you selected other, please describe here.

________________________________________________________________

6. Is your hospital a trauma center?

- Yes
- No

7. Level of trauma center?

- Level I
- Level II
- Level III
- Level IV
- Level V

8. What is the estimated annual patient volume of your ED?

- Under 10K
- 10K-50K
- 50K+

9. Geographic location

- Northeast
- Southeast
- Midwest
- Southwest
- West
- South

10. Number of hospital beds?

- Under 100
- 100-500
- 500+

11. How well do you think the current staffing plan aligns with the demands of the ED, considering patient volume and acuity? Rank on a scale of 1(not well) to 5 (very well).

12. How effective are the current procedures for shift handovers in ensuring the continuity of patient care? Rank on a scale of 1 (very ineffective) to 5 (very effective).

13. Does your ED use protocols in triage?

- Yes
- No

14. Rank the following 1-5 in order of need to improve overall patient flow across your ED

- Simple and expedited registration process to reduce wait time…..
- Optimize use of physical space……
- Use of virtual technology/ AI/machine learning to improve triage and patient care…..
- Appropriate care management resources…..
- Better communication systems to enhance collaboration among ED team…..

15. How satisfied are you with the on-boarding and orientation processes for new staff in the emergency department?   Rank on a scale of 1 (very dissatisfied) to 5 (very satisfied).

16. How often do you encounter issues related to denied claims or coding errors in the emergency department's revenue cycle?

- Very rarely
- Rarely
- Occasionally
- Often
- Very often
- Unknown

17. What, in your opinion, would be the most significant improvement that could enhance the overall patient experience in the ED? (Short response answer)

18. Would you be open to participating in a follow-up interview or providing additional feedback to further explore your experiences and suggestions?

- Yes
- No

19. Kindly share your email address.

________________________________________________________________
