# Supplementary material for: Operational challenges and adaptive leadership in emergency departments in the United States of America: a mixed-methods analysis
Source: BMC Emerg Med. 2025 Nov 19;25:241. doi: 10.1186/s12873-025-01400-y (PMC12628554; doi:10.1186/s12873-025-01400-y)
Supplement: Supplementary file 2 — Supplementary Material 2 [file 12873_2025_1400_MOESM2_ESM.docx]

**In-depth Interview Guide**

**Study Title:** Powering ED Performance

| Begin with the demographics form. Then start recording.  Record following when you begin recording this interview:  a. Date and starting time of interview  b. Participant ID |
| --- |

**Introduction (2-3 minutes)**

*Thank you for agreeing to participate in this follow-up interview. We appreciate your time and insights. This conversation aims to better understand the operational challenges and leadership practices within emergency departments across the U.S. Your responses will remain confidential and will be used solely for research purposes to help identify patterns and recommend evidence-based strategies for improving emergency department performance.*

*Do you have any questions before we begin?*

*May I start the recording?*

*I would now like to start the interview by asking you to speak a little bit about yourself.*

**I. Leadership Context**

1. Can you tell me about your current role and how long you've held this position?
2. What do you see as the biggest day-to-day leadership challenge in your ED?

**II. Operational Challenges**

1. How would you describe your current ED staffing structure? Are there particular workforce pressures you're facing (e.g., turnover, scheduling gaps)?
2. Are there specific bottlenecks in your patient flow? If so, where do they tend to occur most frequently?
3. What recent strategies or interventions have you tried to address these challenges? How effective have they been?

**III. Technology and Systems**

1. Does your ED use any decision-support tools, virtual care platforms, or AI solutions? If yes, how have these affected your operations and outcomes?
2. How do you think technology could be better integrated into ED processes to support staff and improve patient outcomes?

**IV. Leadership and Team Dynamics**

1. How would you describe the communication and coordination among ED team members-nurses, physicians, support staff, etc.?
2. What kinds of onboarding or leadership training are available for new ED staff or managers? Are there gaps you wish were addressed?

**V. Policy and Financing**

1. Do you experience challenges related to billing, coding, or reimbursement in your ED operations?
2. What policy changes at the hospital or state level do you believe would most help your ED run more effectively?

**VI. Final Reflections**

1. What one change—if resourced and supported—do you think would most improve your emergency department’s performance?
2. Is there anything else you'd like to share that we haven't yet covered?

*Thank you so much for sharing your thoughts. Your input is extremely valuable to our analysis and will help us produce more targeted and actionable insights. We may reach out for clarifications if needed. Have a great day!*
